# Trunk Injection for Arthropod Pest Management in Woody Plants: A Comparative Perspective Under Greenhouse and Field Conditions

**DOI:** 10.3390/plants15101481

**Published:** 2026-05-12

**Authors:** Marius Paraschiv

**Affiliations:** National Institute for Research and Development in Forestry “Marin Drăcea”—Brasov Station, Closca Street, 13, RO50040 Brasov, Romania; marius.paraschiv@icas.ro

**Keywords:** trunk injection, plant–insect interactions, woody plants, systemic insecticides, pest management, canopy distribution

## Abstract

Woody plants in managed and natural ecosystems are increasingly exposed to arthropod pest pressure, posing challenges for sustainable plant protection. This study evaluates trunk injection as a pest management strategy in woody species, with emphasis on plant-mediated processes shaping interactions between host plants and herbivorous arthropods. Two experimental systems were investigated: a greenhouse experiment with *Gleditsia triacanthos* and a field experiment with *Quercus petraea*. Systemic active ingredients (acetamiprid and abamectin) were applied using both experimental and professional injection devices, and their effectiveness was assessed against *Tetranychus urticae* and a complex of foliar-feeding insects. In the greenhouse experiment, trunk injection reduced *T. urticae* populations compared with untreated controls and soil drench treatments, with reductions of 55.6–58.4% for larvae, 65.7–67.5% for eggs, and 28.7% for adults, although foliar application achieved higher suppression (up to 81.2% for eggs). In the field experiment, treatments reduced leaf discoloration (from 16.3% in control to 1.45–3.80%) and skeletonization (from 15.1% to 9.95–12.7%), with more moderate effects on defoliation. Differences among feeding guilds suggest that responses to systemically distributed compounds depend on feeding behavior and canopy position. Both pressurized and non-pressurized methods enabled uptake and translocation of active compounds within plant tissues. Localized injuries were observed at injection points, including internal necrosis. Overall, trunk injection represents a viable approach for pest management in woody plants, highlighting the role of plant-mediated processes in shaping treatment outcomes under contrasting conditions.

## 1. Introduction

Systemic protection in woody plants depends on the movement of water and dissolved active ingredients through the vascular system. In trees, upward transport through the xylem is influenced by the physicochemical properties of the compound as well as by species-specific anatomy, physiological activity, and environmental conditions regulating sap flow and transpiration [1,2,3]. Under these conditions, pesticide efficacy may reflect the efficiency of internal transport processes as much as the intrinsic toxicity of the active substance [4].

Although still debated [5,6], long-distance xylem transport is generally explained through cohesion theory, which attributes sap ascent to tension generated by leaf evapotranspiration [7,8]. Woody species differ markedly from herbaceous plants in structural and functional terms, with extensive vascular networks and spatially variable physiological activity within the canopy [9,10,11,12]. Transport dynamics are closely linked to water availability and seasonal patterns, but also to stand-level variation in radiation, temperature, air humidity, and vapor pressure deficit [13,14,15]. These variables can alter both compound distribution and biological response, making host characteristics and local environmental conditions central to treatment performance.

Trunk injection introduces pesticides or nutrients directly into the vascular tissue. By avoiding spray applications or soil drench, external exposure is reduced and off-target contamination becomes less likely [16,17]. In practice, however, tree species traits, environmental variability, and pest pressure do not act independently, and their combined influence may contribute to variability in treatment outcomes across different experimental conditions.

Injections are usually described as passive or active [18]. Passive injections rely on natural uptake from a reservoir connected to the vascular system, while active injections deliver the compound into the xylem under applied pressure. The choice depends on tree size and physiological status. Techniques range from localized micro-injections to multiple injection ports designed for larger volumes. These technical differences affect the uptake rate and internal distribution. In addition, the creation of injection ports represents a localized mechanical injury that may affect tissue integrity and potentially influence subsequent transport processes [19]. Experimental work has shown that applied pressure, number and diameter of drill holes, and port placement can substantially modify treatment outcomes [20,21].

The tree’s physiological condition at the time of application is another determining factor. Transpiration rate, water status, cambial activity, and phenological stage influence compound movement. Identical procedures may therefore produce different results across species, seasons, or environmental contexts. Also, vessel anatomy constitutes a crucial factor, as previous studies have demonstrated that ‘injectability’ differs among tree species depending on the anatomical characteristics of their vascular tissue [22].

Ecological complexity differs between experimental systems. Greenhouse conditions reduce abiotic variability and limit biological interactions [23], resulting in simplified host–pest structures. Field systems are inherently more heterogeneous. Environmental fluctuations, variation among trees [24], and naturally developing pest populations introduce multiple interacting factors. Evaluating injection efficacy under these contrasting scenarios allows a better understanding of how treatment outcomes vary under different environmental and biological contexts.

Pest guild structure adds another layer of variability. Herbivorous arthropods differ in feeding strategy, from cell-sap feeders such as aphids to defoliators and leaf miners that consume or degrade foliar tissue [25,26,27]. These differences can influence both exposure duration and dose at the feeding site, affecting the biological response to delivered compounds. Considering different functional groups provides additional context for interpreting responses to trunk-injected compounds under varying trophic conditions.

Physiological, ecological, and trophic factors interact rather than acting independently, and their combined effects may generate variability that is difficult to anticipate. This raises a practical question regarding the stability of results obtained in simplified systems once treatments are applied under field conditions.

Despite the expanding use of trunk injection in pest management, evaluations conducted under controlled and field conditions are often reported separately, and their outcomes are rarely interpreted within a common conceptual context. Many studies have focused either on controlled experiments involving a single species or on field applications lacking a corresponding controlled reference [28,29,30]. Previous studies have typically addressed trunk injection either under controlled conditions or in field applications, often focusing on single species or specific pest systems, or have approached the method from a general conceptual perspective [17,18]. As a result, the extent to which treatment performance remains consistent across contrasting ecological conditions is still insufficiently understood.

In this study, plant-mediated processes refer to the physiological mechanisms governing the uptake, transport, and redistribution of systemically applied compounds within plant tissues. In this context, transpiration-driven xylem flow represents a key mechanism influencing the efficiency of pesticide movement and its distribution across canopy positions and feeding sites.

To address this gap, trunk injection efficiency was assessed in two woody species representing distinct experimental contexts. Under controlled greenhouse conditions, treatments were applied to honey locust (*Gleditsia triacanthos*) and evaluated against the two-spotted spider mite, *Tetranychus urticae*, a sap-feeding pest typical of protected environments [31,32], based on population density assessments. Field applications targeted sessile oak (*Quercus petraea*) subjected to natural infestation by a heterogeneous pest assemblage, including defoliators, sap feeders, and leaf miners, with treatment performance evaluated through visible foliar injury. These systems differ in ecological complexity and pest structure, providing insight into treatment responses under contrasting biological and environmental conditions. The study aimed to evaluate treatment performance across these distinct experimental designs and to assess how pest feeding guilds and ecological context may influence treatment outcomes under differing experimental conditions. These pest systems represent both model organisms under controlled conditions and ecologically relevant pests of woody species in natural environments, allowing the evaluation of treatment performance across different biological contexts.

## 2. Results

### 2.1. Trunk Injection Experiments in Honey Locust (Gleditsia triacanthos)

The biometric characteristics of the experimental plants are presented in Table 1. No significant differences were detected among treatments in plant height (Kruskal–Wallis test, χ^2^ = 0.14, df = 3, *p* = 0.987), height to the first branch (χ^2^ = 1.80, df = 3, *p* = 0.616), or stem diameter (χ^2^ = 4.29, df = 3, *p* = 0.232). These results indicate that the plants used in the experiment were comparable in size across treatments. Overall, the experimental plants averaged approximately 161.91 ± 14.88 cm in height, with the canopy beginning at about 47.78 ± 11.13 cm above the soil.

#### 2.1.1. Effects of Treatments Across Developmental Stages of *T. urticae*

**Figure 1:**
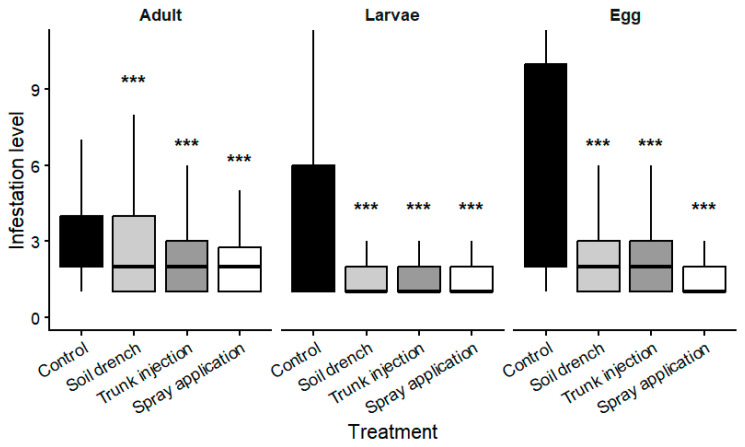
Infestation levels (individuals per leaf) recorded for adults, larvae, and egg stages under different treatments. Values represent mean ± SD. Asterisks indicate significant differences compared with the untreated control based on Dunn’s post hoc test following Kruskal–Wallis (Bonferroni-adjusted *p*-values; *** *p* < 0.001).

Significant differences among treatments were detected for all analyzed developmental stages of *T. urticae* (Kruskal–Wallis test: adults, χ^2^ = 166.2, df = 3, *p* = 9.64 × 10^−36^; larvae, χ^2^ = 162.4, df = 3, *p* = 8.41 × 10^−35^; eggs, χ^2^ = 850.1, df = 3, *p* = 7.66 × 10^−184^). Mean densities recorded under each treatment are presented in Table 2 and illustrated in Figure 1.

These values correspond to reductions of approximately 55.6–58.4% for larvae, 65.7–67.5% for eggs, and 28.7% for adults relative to the untreated control.

Across all developmental stages (larvae, eggs, and adults), untreated plants consistently showed higher population densities compared with treated plants. All treatments reduced larval densities relative to the control, with only minor differences observed among application methods. In contrast, egg densities showed clearer differences among treatments, with the strongest reduction observed under foliar application, followed by trunk injection and soil drench.

Adult densities showed a similar pattern, remaining consistently lower in treated plants than in untreated controls. Overall, differences among treatments were less pronounced for larvae and more evident for eggs and adults.

Within the trunk injection treatment, no significant differences were observed between injection orientations (parallel vs. perpendicular) across developmental stages (Mann–Whitney U test, *p* > 0.05). Mean infestation levels were similar between orientations, with values of 2.26 ± 1.54, 1.80 ± 1.15, and 2.46 ± 2.21 (adults, larvae, and eggs) for parallel injections, compared with 2.34 ± 1.51, 1.76 ± 1.14, and 2.52 ± 2.18 for perpendicular injections. These results indicate that injection direction did not influence infestation levels.

#### 2.1.2. Vertical Distribution of *Tetranychus urticae* Within the Canopy

**Figure 2:**
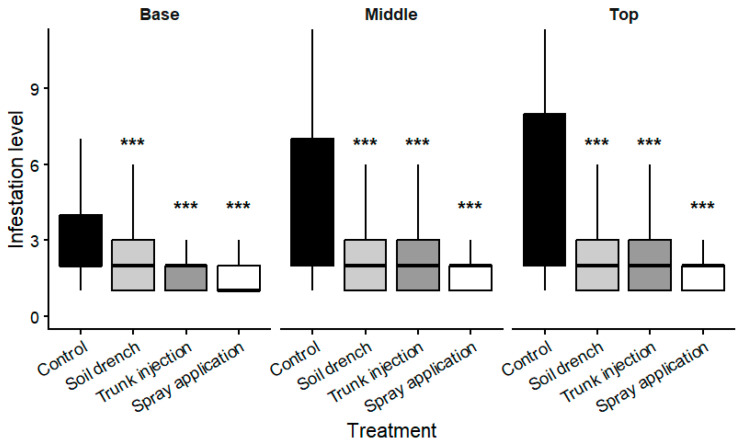
Infestation levels (individuals per leaf) recorded at different canopy positions (base, middle, and top) under different treatments. Values represent mean ± SD. Asterisks indicate significant differences compared with the untreated control based on Dunn’s post hoc test following Kruskal–Wallis (Bonferroni-adjusted *p*-values; *** *p* < 0.001).

Significant differences among treatments were detected at all canopy positions (Kruskal–Wallis test: base, χ^2^ = 309.0, df = 3, *p* = 8.74 × 10^−67^; middle, χ^2^ = 331.0, df = 3, *p* = 1.51 × 10^−71^; top, χ^2^ = 397.0, df = 3, *p* = 8.94 × 10^−86^) (Table 3; Figure 2).

In the control, densities increased markedly from the base to the upper canopy, indicating a clear vertical gradient in mite distribution. In contrast, treated plants showed more stable infestation levels across canopy positions, suggesting a more uniform distribution.

Reductions relative to the control were observed at all canopy levels and were generally more pronounced in the middle and upper canopy. Differences among treatments became more evident at the upper canopy level, where foliar application and trunk injection maintained lower infestation levels compared with soil drench, with similar but less pronounced patterns observed at the base and middle canopy. These patterns were supported by post hoc comparisons, which confirmed significant differences between treated and untreated plants at each canopy level.

No significant differences were observed among canopy positions within the trunk injection treatment (Mann–Whitney U test, *p* = 0.084). Mean infestation levels were similar across canopy positions for both injection orientations, with only minor variation between the base, middle, and upper canopy. For parallel injections, values ranged from 1.96 ± 1.39 at the base, 2.34 ± 1.90 in the middle canopy, and 2.40 ± 2.00 at the top, while comparable values were recorded for perpendicular applications (2.09 ± 1.40, 2.21 ± 1.70, and 2.53 ± 2.12). These results indicate a relatively uniform distribution of infestation levels within the canopy, regardless of injection orientation.

### 2.2. Trunk Injection Experiments in Oak (Quercus petraea)

Tree diameter did not differ significantly among treatments (Kruskal–Wallis test, χ^2^ = 4.50, *p* = 0.105), with mean values of 16.93 ± 1.78 cm for the single-injection treatment, 17.43 ± 2.49 cm for the double-injection treatment, and 18.03 ± 1.79 cm for the control.

Similarly, the height to the first branch showed moderate variation, with mean values of 6.62 ± 1.54 m (20 mL), 7.17 ± 2.67 m (40 mL), and 7.47 ± 1.14 m (control). However, these differences were not statistically significant (Kruskal–Wallis test, χ^2^ = 5.59, *p* = 0.06).

The absence of significant differences in both diameter and height to the first branch suggests that trees were structurally comparable across treatments prior to injection.

#### 2.2.1. Leaf Damage Indicators

**Figure 3:**
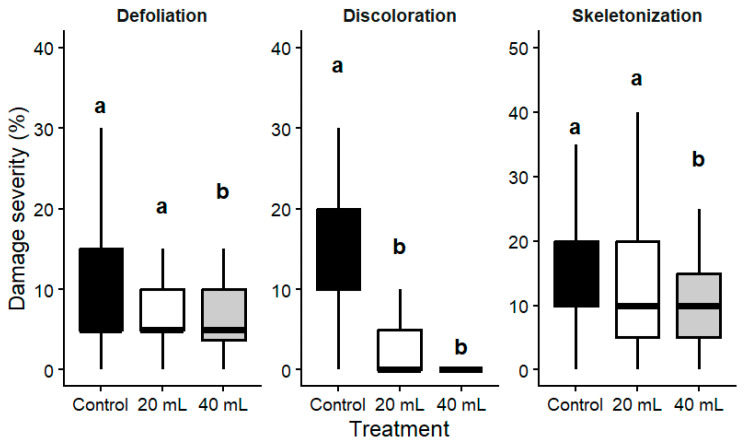
Effects of trunk injection treatments on leaf damage parameters (defoliation, discoloration, and skeletonization) in *Q. petraea*. Boxplots represent median values, interquartile ranges, and whiskers indicating minimum and maximum values. Different letters indicate significant differences among treatments based on Dunn’s post hoc test following the Kruskal–Wallis test (Bonferroni-adjusted *p* < 0.05).

Significant differences among treatments were detected for all analyzed leaf damage indicators (defoliation, χ^2^ = 13.0, df = 2, *p* = 1.52 × 10^−3^; discoloration, χ^2^ = 157.0, df = 2, *p* = 9.17 × 10^−35^; skeletonization, χ^2^ = 17.5, df = 2, *p* = 1.60 × 10^−4^ (Table 4; Figure 3).

The reduction was most pronounced for discoloration, where treated trees showed minimal symptoms, while untreated trees exhibited substantially higher levels. Skeletonization showed a more moderate response, with significant reductions observed only at the higher dose, whereas the lower dose did not differ significantly from the control. In contrast, defoliation showed a more limited response to treatment, with only slight differences among variants.

#### 2.2.2. Presence of Insect Traces on Leaves

**Figure 4:**
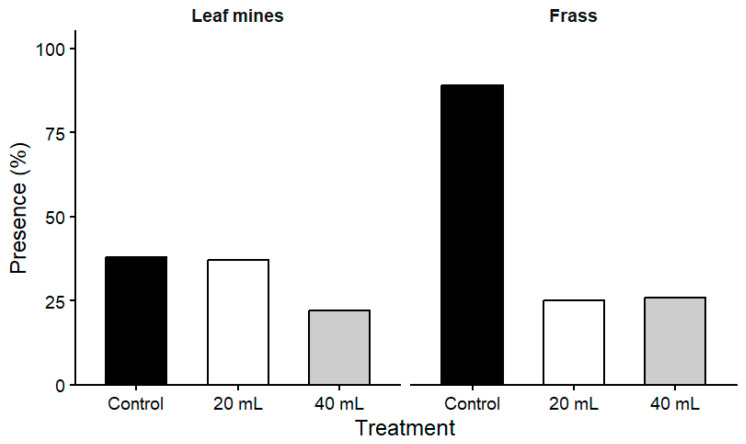
Presence of leaf mines and frass under different treatments. Bars represent the percentage of leaves exhibiting mines or frass for each treatment. Presence was recorded as a binary variable (presence/absence).

The proportion of leaves exhibiting insect traces (frass and leaf mines) varied between pest types (Table 5; Figure 4). Significant differences among treatments were detected for frass presence (Kruskal–Wallis test, χ^2^ = 108.0, df = 2, *p* = 4.17 × 10^−24^), whereas no statistically significant differences were observed for leaf mines (χ^2^ = 5.76, df = 2, *p* = 5.61 × 10^−2^).

The presence of frass attributed to *C. arcuata* was markedly reduced in treated trees compared with the control, indicating a clear treatment effect. In contrast, the proportion of leaves showing mines did not differ significantly among treatments, suggesting a limited response of leaf-mining insects to the applied treatments.

### 2.3. External Lesions and Internal Stem Tissue Responses Following Trunk Injection

In honey locust seedlings, external lesions were observed at the injection points in treated individuals. Visible symptoms were observed in 21 out of 30 seedlings (70%), while no external symptoms were detected in the remaining 9 seedlings (30%).

**Figure 5:**
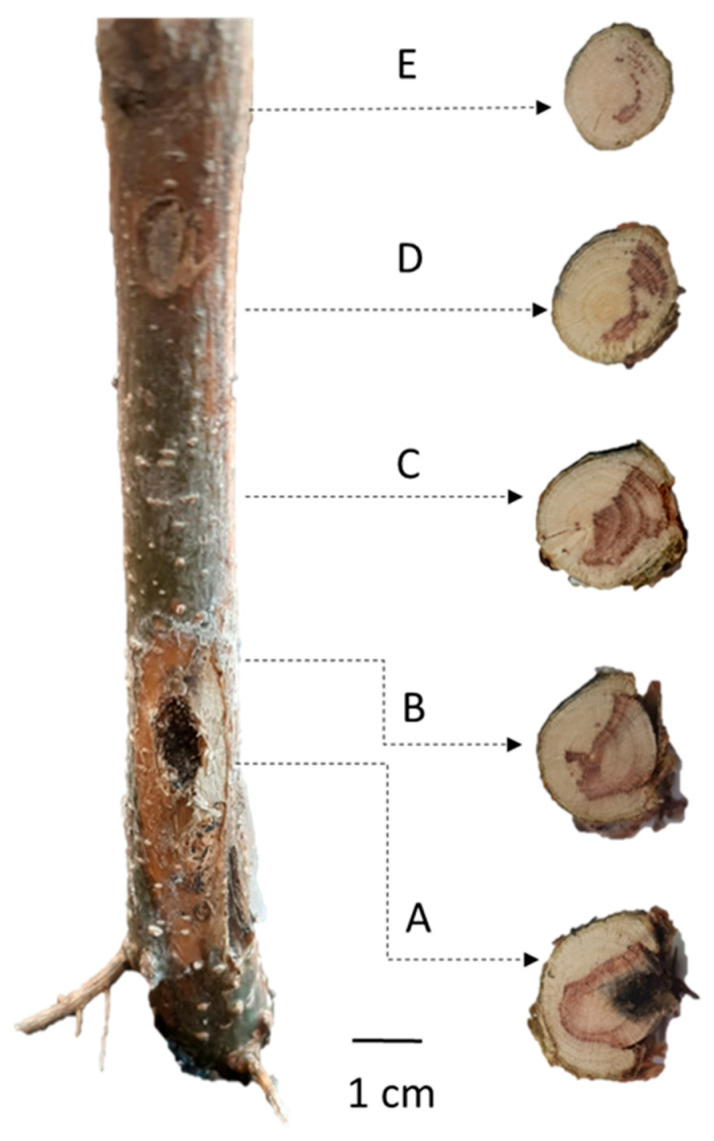
Transverse stem sections of *G. triacanthos* showing internal necrosis at different vertical positions relative to the injection point: (A) at the injection point, (B) 1 cm above the injection point, (C) 3 cm above, (D) 5 cm above, and (E) 7 cm above. The images illustrate the spatial extent and compartmentalization of tissue damage following trunk injection.

These lesions were generally small in size, with a mean length of 2.54 ± 1.78 cm and a mean width of 0.79 ± 0.66 cm (Figure 5). They consisted primarily of bark discoloration (brownish) and localized drying of the outer bark.

**Figure 6:**
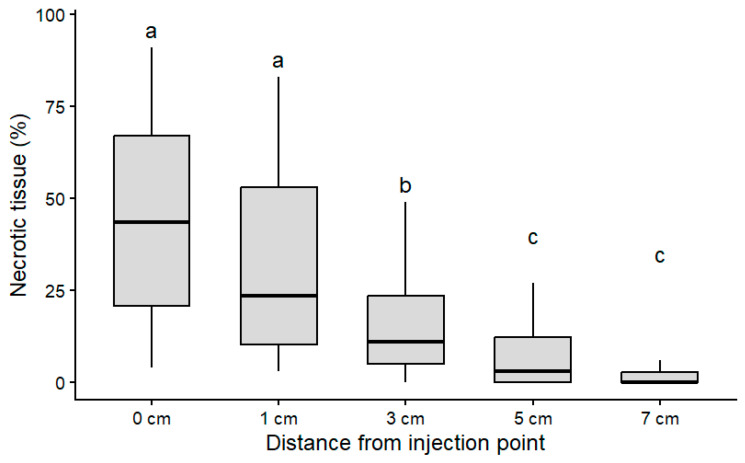
Percentage of necrotic tissue in stem cross-sections at different distances from the injection point (0, 1, 3, 5, and 7 cm) in *G. triacanthos*. Boxes represent median values, interquartile range, and whiskers indicating the full range of observed values. Different letters indicate significant differences in necrotic tissue proportion among stem positions according to Dunn’s post hoc test (*p* < 0.05).

Internal tissue necrosis was detected in cross-sections taken along the stem above the injection point in all examined plants (30/30). The proportion of necrotic tissue varied significantly among the examined positions (Kruskal–Wallis test, χ^2^ = 76.63, df = 4, *p* = 9.00 × 10^−16^). The highest proportion of necrotic tissue was recorded at the injection point (44.9 ± 27.5%), remaining relatively high at 1 cm above it (35.5 ± 25.4%). Necrosis decreased markedly at 3 cm above the injection point (14.7 ± 14.7%), while substantially lower values were observed at 5 cm (8.3 ± 10.0%) and 7 cm (3.2 ± 6.7%). Pairwise comparisons using Dunn’s test confirmed significant differences between the injection point and the positions located 3–7 cm above it (Figure 6).

In the field experiment, in the double-injection treatment (40 mL), lesion length differed significantly between upslope and downslope sides of the trunk, with mean values of 7.41 ± 4.70 cm and 4.16 ± 2.12 cm, respectively (paired Wilcoxon signed-rank test, *p* = 0.011).

Upslope lesion length averaged 4.34 ± 2.77 cm in the 20 mL treatment and 7.41 ± 4.70 cm in the 40 mL treatment. Although lesions tended to be larger in the double-injection treatment, this difference did not reach statistical significance (Mann–Whitney U test, *p* = 0.054).

## 3. Discussion

Our study evaluates the potential of trunk injection as a method for controlling forest pests under both field and greenhouse conditions, while foliar spray and soil drench were included exclusively in the greenhouse experiment to provide a comparative framework for application methods. In both experimental systems, pest pressure was reduced, suggesting that treatment efficacy was influenced by processes governing the uptake, transport, and redistribution of systemically delivered insecticides within plant tissues. These observations indicate that treatment outcomes are not only a function of the active compound, but also of how compounds move through the xylem under the influence of transpiration-driven flow and plant architecture, particularly under contrasting conditions of canopy size, plant structure, and environmental variability.

In our experiment, the strongest effect on *T. uricae* was observed at the larval stage compared with adults and eggs. The relatively uniform reduction in larval densities across treatments is consistent with similar exposure patterns of mobile stages to active compounds. In contrast, the greater variability observed for eggs and adults may reflect indirect effects, as eggs are not directly exposed to the insecticide and their abundance depends on adult survival and oviposition behavior. These responses are also consistent with the mode of action of abamectin, which interferes with neural transmission in arthropods, leading to paralysis and mortality, with its effectiveness depending on redistribution within plant tissues and availability at feeding sites. This stage-specific response suggests that treatment efficacy is closely linked to the accessibility of the compound at the feeding interface, which is influenced by both pest mobility and the spatial distribution of the active ingredient within leaf tissues.

The two experimental systems differed substantially in ecological complexity, with greenhouse conditions providing a simplified environment with homogeneous plant structure and controlled abiotic factors, whereas field conditions introduced greater variability in canopy architecture, microclimate, and pest assemblages. As a result, treatment responses should be interpreted within their respective contexts rather than as directly comparable outcomes across systems. Although the two experiments differed substantially in scale and ecological complexity, trunk injection reduced *T. urticae* populations in the greenhouse and decreased foliar damage in field-grown trees. However, responses were more consistent under controlled conditions and more variable in the field, indicating experiment-dependent outcomes.

The absence of differences between densities recorded on seedlings injected parallel and perpendicular to the first branch, together with the similar infestation levels observed across canopy positions, suggests a relatively uniform pattern of infestation at the scale of the experimental seedlings, without implying uniform distribution of the active compound. Given the small size of the seedlings (<2 m) and the short distance between the injection point and the canopy (<0.7 m), translocation appears to have been effective at the scale of the experimental seedlings, likely due to reduced path length and limited hydraulic resistance within the xylem network. In this context, the limited transport distance likely facilitated rapid redistribution of the active compound within the crown, contributing to relatively uniform internal concentrations. This contrasts with mature trees, where transport pathways are longer and more complex, as well as with species showing sectorial distribution patterns [33,34].

In untreated plants, infestation tended to increase toward the upper canopy, likely reflecting differences in leaf traits or microenvironmental conditions. In treated plants, this gradient was less pronounced, indicating that treatments modified the vertical distribution of infestation through internal redistribution of active compounds. Trunk injection was associated with reduced variation in infestation levels across canopy positions compared with the control. This pattern may reflect a reduction in the natural infestation gradient rather than a truly homogeneous distribution of the active compound within the canopy. These observations are limited to the scale of the experimental seedlings and should not be extrapolated beyond this context.

In the greenhouse experiment, foliar application resulted in higher efficacy than trunk injection, and higher efficacy than soil drench, likely reflecting differences in uptake pathways rather than intrinsic performance. Foliar application ensures direct contact and better coverage, whereas soil drench relies on root uptake and subsequent transport, which may be limited or temporally delayed under the experimental conditions used, particularly given the constrained root volume and controlled irrigation regime. From a physiological perspective, foliar application provides immediate deposition on leaf surfaces and rapid penetration into epidermal tissues, whereas trunk injection depends on upward xylem transport driven by transpiration, and soil drench requires root absorption followed by loading into the vascular system, a process that may be slower and more variable. Similar responses have been reported for systemic insecticides such as imidacloprid, where trunk injection enables more direct and rapid translocation, whereas soil-based applications are more dependent on environmental conditions affecting root uptake [35,36]. Limited transport following root absorption cannot be excluded, as distribution may occur primarily through vessels formed in the current annual growth and may require longer periods for effective uptake [17,37]. This may partially explain the observed results. Nevertheless, population densities remained lower than in the control, indicating that internal redistribution can still contribute to pest suppression.

In our field experiment on sessile oak, trunk injection with acetamiprid was associated with reduced damage levels compared with the control, with differences depending on the applied dose, consistent with findings reported in fruit crops [38]. This trend is consistent with previous studies reporting high efficacy of trunk-injected acetamiprid against foliar pests, indicating that sufficient systemic distribution can be achieved under field conditions. Differences among damage types are likely related to feeding behavior and to the temporal dynamics of exposure within the canopy of mature trees. Defoliation, caused by lepidopteran larvae, showed a more limited reduction, likely because feeding continues until a lethal dose is ingested. In contrast, mobile adults responsible for leaf discoloration may interrupt feeding and migrate once the compound is detected. These patterns indicate that treatment effectiveness depends on both the duration of feeding required to reach a lethal dose and the capacity of insects to avoid or escape exposure. These differences further support the role of feeding guild, as insects with distinct feeding strategies interact differently with the spatial distribution of systemically delivered compounds. Accordingly, a guild-based approach was adopted, reflecting common practice in forest systems where pest complexes are evaluated at the functional level rather than through detailed species-level quantification.

In our study, leaf-mining insects did not show a clear treatment-related response, despite their limited mobility. This may indicate that the timing and distribution of the active compound were insufficient to consistently influence oviposition or larval establishment. Given the absence of significant differences in tree size among treatments, these results are more likely related to variability in compound distribution within the canopy rather than differences in dose allocation at the whole-tree level. A similar pattern was observed for *C. arcuata*, where the presence of frass was markedly reduced in treated trees, while leaf mining incidence remained relatively unaffected, suggesting differences in exposure associated with feeding strategy. In addition, the endophytic feeding niche of leaf miners may limit effective exposure to systemically distributed compounds, as these are transported primarily through vascular tissues and may not reach sufficiently high or homogeneous concentrations within mesophyll tissues where larvae develop.

External lesions at injection points represent a known consequence of trunk injection. Although direct comparison between the two experiments is limited by differences in experimental conditions, external lesions were of comparable magnitude in both systems, generally extending a few centimeters around the injection point. Despite differences in tree size and injection method, this suggests that localized mechanical injury is a consistent consequence of trunk injection, although its physiological impact may differ depending on species and stem structure. Such injuries may facilitate pathogen entry into woody tissues; however, their progression depends on severity, and they may be partially or completely healed over several growing seasons, as previously reported [39].

Internal necrosis observed in honey locust was sometimes extensive, occasionally affecting a substantial portion of the stem cross-section, but remained spatially limited, with evidence of compartmentalization restricting its vertical spread to a few centimeters from the injection point. This response is consistent with previous findings indicating a relatively strong compartmentalization capacity in honey locust [40], which may contribute to limiting tissue damage.

In sessile oak, differences in lesion size between injection positions may be related to uneven distribution of the injected solution within the stem. The position of the injection point on sloped terrain (upslope vs. downslope) may influence local dispersion of the solution, resulting in uneven exposure of surrounding tissues. In addition, the upward movement of xylem sap may promote higher concentrations above the injection point, which could explain the larger lesions observed in those areas.

Taken together, these findings indicate that treatment efficacy is not solely determined by the active compound, but by the interaction between compound properties, transport distance, tree architecture, and environmental conditions.

The main limitation of this study is the lack of quantification of active compound concentrations within plant tissues (i.e., residue analysis), which may vary considerably [41] and prevents direct assessment of compound distribution and limits mechanistic interpretation of treatment efficacy. In addition, no solvent-only injection control was included, which restricts the ability to fully separate potential mechanical effects associated with trunk injection from the effects of the active substances. Furthermore, pest assessment in the field experiment was conducted at the level of feeding guilds, and species-level identification was not systematically performed. This limits the taxonomic resolution of the results and constrains ecological interpretation of pest-specific responses. Evaluations were limited to a single growing season, and tree-to-tree variability and environmental heterogeneity may also have influenced treatment responses [42,43]. In addition, the interpretation of distribution patterns is constrained by the scale of the experiments, as results obtained on seedlings cannot be directly extrapolated to mature trees with more complex vascular architecture. These limitations should be considered when interpreting the results, as the observed patterns reflect responses under specific experimental conditions and should not be generalized beyond the scope of the present study.

Future studies should investigate higher doses, alternative application timings, and additional systemic insecticides, as these factors may influence treatment performance. Similar aspects have been highlighted in previous studies [18,44]. Expanding the range of tested compounds is particularly relevant, given that the development of injection technologies currently outpaces the availability of active substances suitable for this application [45]. In addition, the possibility of automating injection procedures should be considered, as suggested by recent studies [46].

In summary, the present findings indicate that trunk injection can contribute to pest suppression in woody species under contrasting experimental conditions, although its performance depends on the interaction between plant physiological processes, environmental conditions, and pest functional traits. These findings further support the potential of trunk injection as a more precise, targeted, and environmentally controlled approach to pest management in such systems.

## 4. Materials and Methods

### 4.1. Study Site and Experimental Design

Two experiments were conducted to evaluate trunk injection treatments in contrasting conditions: a controlled greenhouse experiment and a field experiment under forest conditions.

#### 4.1.1. Greenhouse Experiment (*Gleditsia triacanthos*)

The greenhouse experiment was conducted under controlled conditions at the National Institute for Research and Development in Forestry, using honey locust (*G. triacanthos*) as the host species. Four-year-old potted plants were maintained under regulated temperature and humidity conditions, allowing the isolation of experimental units from major environmental variability.

Plants were grown in 15 L pots containing a substrate composed of a commercial organic growth medium, peat, and perlite (4:2:1, *v/v/v*). Environmental conditions were maintained within a temperature range of 10–35 °C, with relative humidity kept above 40% through periodic irrigation. Plants were exposed to natural daylight conditions throughout the experimental period. To minimize positional effects within the greenhouse, pots were arranged randomly and repositioned at regular intervals (every 7–10 days) throughout the experimental period.

The experiment focused on a single pest species, the two-spotted spider mite *T. urticae* (Tetranychidae, Trombidiformes). The mite population was naturally established in the greenhouse and had persisted for several years prior to the experiment, resulting in a consistent background infestation across multiple growing seasons.

In the greenhouse experiment, the experimental design included three treatment variants (trunk injection, foliar spraying, and soil drenching) and an untreated control, with no injections performed using water or solvent-only solutions. Each treatment consisted of three replicates of ten plants (*n* = 30 per treatment; *n* = 30 for the control). Plants were randomly assigned to treatments. The density of *T. urticae* at the leaf level was used as the observational variable, while individual plants were considered the experimental units, with data aggregated at the plant level prior to analysis. Damage levels across three canopy positions (lower, middle, upper) and developmental stages (eggs, larvae, adults) were included as response variables. In addition, the proportion of necrotic tissue associated with injection points was quantified and included as a response variable. Biometric variables, including total plant height, height to crown base, and stem diameter, were recorded prior to treatment application to ensure comparability among experimental units.

#### 4.1.2. Field Experiment (*Quercus petraea*)

The field experiment was conducted under forest conditions at 45°46′40.57″ N, 25°54′20.47″ E, at an altitude of 550 m a.s.l., using sessile oak (*Q. petraea*) as the host species. The stand had a natural composition, with a canopy cover of approximately 80%. Trees were approximately 60 years old, with a mean diameter at breast height (DBH) of 16–18 cm and an average height of 15–16 m.

The site is representative of typical forest conditions for sessile oak in the region, with an average annual temperature of 7.5 °C and mean annual precipitation of approximately 700 mm. The study area is characterized by the presence of multiple foliar pest groups typically associated with sessile oak stands.

The experiments included two trunk injection treatments (single-dose and double-dose) and an untreated control. A total of 60 trees were randomly selected for treatment, with 30 trees assigned to each treatment variant, while an additional 30 trees served as untreated controls (no injections with water or solvent-only solutions were performed). Trees were selected to ensure comparable size and condition and were spatially distributed across the study area to minimize potential site-related variability. All trees were marked with paint to ensure clear identification of treatments throughout the experimental period.

In the field experiment, defoliation, discoloration, and skeletonization were assessed as quantitative variables, while insect mines and frass were evaluated based on their frequency. Leaves were treated as observational units and trees as experimental units, with all parameters averaged at the tree level prior to analysis. Tree biometric characteristics, including trunk diameter and height to the first branch, were recorded to ensure comparability among experimental units.

### 4.2. Administration of the Treatments

**Figure 7:**
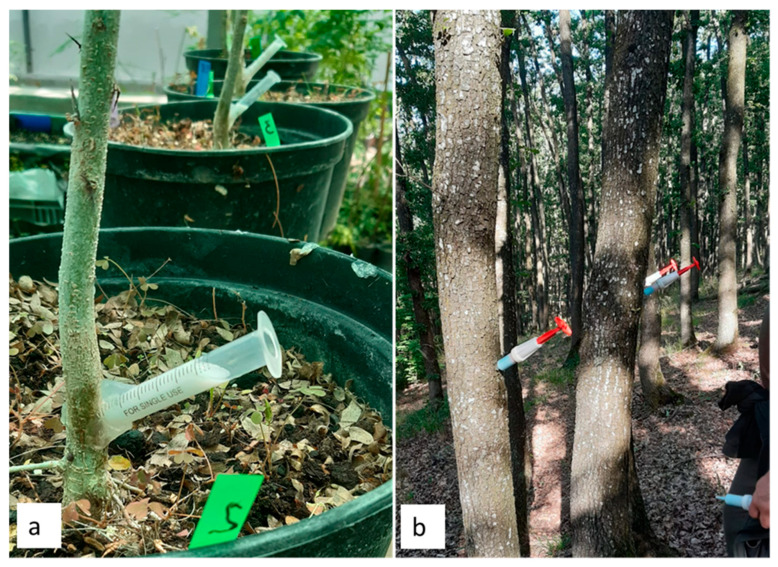
Trunk injection procedure under the experiments. (**a**) Trunk injection was performed on potted honey locust seedlings under controlled greenhouse conditions using a medical syringe. (**b**) Trunk injection applied to mature sessile oak trees under forest conditions using a forestry-specific injector (Chemjet Tree Injector).

In the greenhouse experiment, trunk injection was performed using single-use medical syringes, following preliminary optimization trials (Figure 7). Injection holes were drilled using a handmade drill with a 2.5 mm drill bit at an angle of 45° and to a depth of approximately 5 mm, near the root collar (around 3–5 cm above soil) where the stem diameter was greater. The treated saplings were divided into two groups (*n* = 15 per group). In the first group (Iv1), injections were performed in the direction of the first branch, whereas in the second group (Iv2), injections were applied perpendicular to the direction of the first branch.

The syringe, without a needle, was inserted by clockwise rotation, and the insertion point was immediately sealed with a fast-curing adhesive to prevent leakage and ensure accurate dose delivery. After several minutes, the syringe plunger was removed to depressurize the capsule, allowing passive uptake of the solution without additional pressure.

In the field experiment, injections were performed using a spring-loaded injector (Chemjet Tree Injector), which delivers the solution into the vascular tissue under internally generated pressure. The system operates without external pressure regulation, as the injection force is determined by the device mechanism. Consequently, pressure parameters were not directly measured during application.

Comparative treatments were applied during the same time interval. Foliar spraying was carried out using a hand sprayer over the entire canopy from a distance of 50 cm, targeting the upper leaf surface primarily. The applied volume per plant was controlled by prior calibration of the sprayer reservoir. Soil drenching was performed by applying the insecticide solution around the stem, at approximately 5 cm from the stem base.

All treatments were administered in early June 2025, coinciding with the emergence of the first generation of *T. urticae*. Detailed information on the active ingredients, application methods, and dosages used in the experiment is provided in Table 6. Abamectin (EC) and acetamiprid (SG) commercial formulations were dissolved in water to the target concentrations and applied as aqueous solutions at the specified doses (2.5 mL per plant and 20–40 mL per tree, respectively). The selected doses were based on preliminary trials and existing recommendations for systemic insecticides, aiming to ensure sufficient uptake while minimizing potential phytotoxic effects. The selected active substances were chosen based on their systemic properties and their documented efficacy against key pest groups, with abamectin targeting mite populations and acetamiprid being effective against a broad range of foliar-feeding insects.

### 4.3. Assessment of the Treatment’s Effects

Infestation levels were assessed by counting the number of *T. urticae* individuals per leaf. For each plant, samples were collected from different canopy positions (base, middle, and upper canopy) to ensure representative coverage of the infestation.

Fourteen days after treatments were applied, three leaves (compound leaves consisting of 20–30 leaflets) were collected from each canopy position of each plant, placed individually in paper envelopes, and sealed. Samples were subsequently stored in a freezer until laboratory analysis.

In the laboratory, the contents of each envelope were examined under a stereomicroscope (Stemi 508, Carl Zeiss, Oberkochen, Germany), and the number of individuals was recorded at the leaf level for all developmental stages (eggs, larvae—including nymphal stages—and adults).

To assess tissue responses and injury development 180 days after injection, trunk sections were collected by cutting stems at the injection point and at 1, 3, 5, and 7 cm above it. Discoloration and the extent of affected tissue were quantified by scanning the sections using WinFOLIA (Regent Instruments Inc., Quebec City, QC, Canada), which allowed the calculation of the percentage of affected stem area. Wood condition was further evaluated through manual probing of necrotic tissues using a fine needle, and their mechanical resistance was qualitatively compared with that of apparently healthy tissues from untreated control trees. External lesions were also assessed by measuring their height, providing complementary information on tissue alterations associated with trunk injection.

In the field experiment, treatment efficacy was evaluated at the end of the vegetation season (early September), when the effects of the injected substances were expected to be fully expressed and the activity of major pests had largely ceased. From each cardinal point, one branch was sampled from five trees per treatment, using telescopic pruning shears, resulting in a total of 20 branches per treatment. For each treatment, 400 leaves were randomly selected from the collected branches (100 leaves per cardinal point) and taken to the laboratory for further analysis.

**Figure 8:**
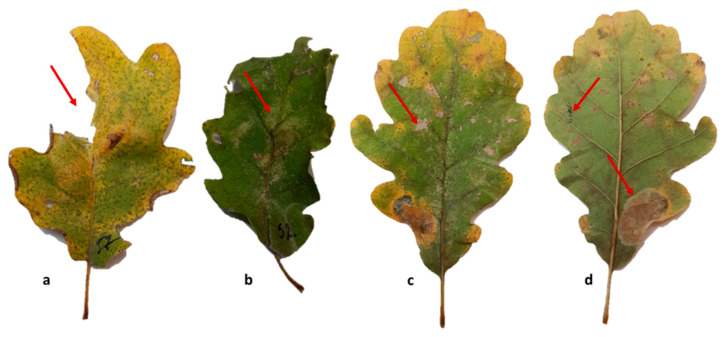
Representative types of leaf damage observed in *Q. petraea*. Arrows indicate the main types of foliar injury: (**a**) defoliation, (**b**) discoloration, (**c**) skeletonization, and (**d**) insect mines and frass.

Laboratory assessments focused on foliar damage, with defoliation, discoloration, and skeletonization visually estimated as a percentage of total leaf area according to similar studies [48], while insect mines and excreta (frass) of *Corythucha arcuata* were recorded based on frequency (Figure 8).

The assessment was conducted at the level of feeding guilds; however, the observed damage types are typically associated with taxa such as *Corythucha arcuata* (sap feeders) and leaf-mining species (e.g., *Bucculatrix* spp., *Phyllonorycter* spp.), while defoliation may be attributed to lepidopteran larvae, although species-level identification was not systematically performed. Wound evaluation was conducted 180 days after injection and focused on external lesions around the injection points, as internal assessment would have required destructive sampling. After carefully removing the bark with a knife, the imprints and discoloration visible in the underlying xylem tissue were measured in vertical extent.

### 4.4. Statistical Analyses

All datasets were tested for normality (Shapiro–Wilk) and homogeneity of variances (Levene’s test). As none of the variables met both assumptions (normality and homogeneity of variances), even after log(x + 1) transformation, non-parametric tests were applied.

Differences among treatment groups were assessed using the Kruskal–Wallis test [49], followed by Dunn’s post hoc test for pairwise comparisons with Bonferroni-adjusted *p*-values [50]. Differences between injection orientations (parallel vs. perpendicular) were evaluated using the Mann–Whitney U test [51], with Bonferroni correction applied when multiple comparisons were performed. Paired comparisons (e.g., upslope vs. downslope lesion lengths within the same trees) were assessed using the Wilcoxon signed-rank test.

Statistical significance was set at *p* < 0.05 for all analyses. All statistical analyses and graphical representations were performed in R version 4.3.2 [52], using packages for nonparametric statistical analysis and data visualization. Data are presented as mean ± standard deviation.

## 5. Conclusions

The results of this study indicate that trunk injection can contribute to reducing pest pressure in woody species under both controlled and field conditions, although its effectiveness depends on multiple interacting factors. Treatment performance was influenced by processes governing the distribution of active compounds within plant tissues, as well as by pest feeding behavior and ecological context. While foliar application showed higher efficacy under controlled conditions, trunk injection consistently reduced pest populations and associated damage compared with untreated controls. The findings also indicate that commonly used systemic insecticides, such as abamectin and acetamiprid, can be effectively delivered and redistributed within plant tissues through trunk injection, contributing to pest suppression under different experimental conditions. However, variability in response highlights the importance of plant structure, environmental conditions, and pest characteristics, supporting the potential role of trunk injection as a complementary and operational tool in integrated pest management strategies for woody species, although these findings should be interpreted within the context of a single growing season and the specific experimental conditions of the present study.

## Figures and Tables

**Table 1:** Biometric characteristics of the experimental plants before treatment application. Values are expressed as mean ± SD.

Treatment	Plant Height (cm)	Height to First Branch (cm)	Stem Diameter (mm)
Control	163.1 ± 15.8	49.0 ± 11.0	13.7 ± 2.11
Soil drench	161.3 ± 13.2	48.7 ± 13.8	13.1 ± 2.89
Trunk injection	162.9 ± 14.0	48.6 ± 11.8	13.5 ± 1.44
Foliar spray	160.8 ± 16.6	45.3 ± 7.9	14.3 ± 2.29

**Table 2:** Mean (±SD) densities of *T. urticae* at different developmental stages under different treatments (individuals per leaf).

Treatment	Larvae	Eggs	Adults
Control	4.21 ± 0.19	7.65 ± 0.26	3.21 ± 0.08
Soil drench	1.75 ± 0.05	2.62 ± 0.08	2.76 ± 0.07
Trunk injection	1.78 ± 0.05	2.49 ± 0.07	2.29 ± 0.05
Foliar spray	1.87 ± 0.09	1.44 ± 0.03	2.02 ± 0.05

**Table 3:** Mean (±SD) densities of *T. urticae* at different canopy positions under different treatments (individuals per leaf).

Treatment	Base	Middle	Top
Control	3.47 ± 0.11	5.34 ± 0.21	6.83 ± 0.29
Soil drench	2.31 ± 0.06	2.34 ± 0.07	2.69 ± 0.08
Trunk injection	2.03 ± 0.05	2.27 ± 0.07	2.46 ± 0.08
Foliar spray	1.45 ± 0.03	2.01 ± 0.00	1.89 ± 0.05

**Table 4:** Mean (±SD) values of leaf damage indicators recorded in *Q. petraea* trunk injection treatments.

Treatment	Defoliation (%)	Discoloration (%)	Skeletonization (%)
Control	9.65 ± 0.79	16.30 ± 0.86	15.10
20 mL/tree	7.65 ± 0.56	3.80 ± 0.68	12.70
40 mL/tree	6.35 ± 0.64	1.45 ± 0.29	9.95

**Table 5:** Proportion (%) of oak leaves showing frass of *C. arcuata* and leaf mines under different treatments based on presence/absence recording.

Treatment	*C. arcuata* Frass (%)	Leaf Mines (%)
Control	89.4	38.3
20 mL/tree	25.1	37.3
40 mL/tree	26.2	22.1

**Table 6:** Characteristics of the insecticides used in trunk injection experiments.

Target Pest/Group	Active Ingredient	Mode of Action	Concentration	Applied Volume	Application Method *
*T. urticae*	abamectin	systemic	2%	2.5 mL per plant	Trunk injection
			2%	2 L per pot	Soil drenching
			0.2%	50 mL per plant	Foliar spraying
Foliar insect pests of *Q. petraea*	acetamiprid	systemic	4%	20 mL per tree	Trunk injection
			4%	40 mL per tree	Trunk injection

* The soil drenching volume (2 L per pot) corresponds to an approximate application rate of 10 L m^−2^, selected to allow solution penetration into the upper soil layer, reach the root zone of seedlings, and facilitate uptake of the active compound [47].

## Data Availability

The original contributions presented in this study are included in the article. Further inquiries can be directed to the author.
